# Heterogenicity of testicular histopathology and tubules as a predictor of successful microdissection testicular sperm extraction in men with nonobstructive azoospermia

**DOI:** 10.1097/MD.0000000000010914

**Published:** 2018-06-01

**Authors:** Yang Yu, Qi Xi, Ruixue Wang, Hongguo Zhang, Leilei Li, Ruizhi Liu, Yuan Pan

**Affiliations:** a Center for Reproductive Medicine and Center for Prenatal Diagnosis, First Hospital; b Jilin Engineering Research Center for Reproductive Medicine and Genetics, Jilin University, Changchun, China.

**Keywords:** heterogeneous, microdissection, nonobstructive azoospermia, seminiferous tubules, testicular histopathology

## Abstract

Only a few studies evaluate the presence of spermatozoa intraoperatively. The study aimed to assess whether the heterogenicity of testicular histopathology and seminiferous tubules can predict the outcome of microdissection testicular sperm extraction (micro-TESE) in men with nonobstructive azoospermia (NOA).

The study comprised a retrospective analysis of 94 patients with azoospermia who were referred from 2016 to 2017. Under optical magnification, they were classified into 2 groups based on the diameter of tubules intraoperatively, namely homogeneous tubules and heterogeneous tubules. Postoperatively, patients were divided into 2 groups of heterogeneous histopathology and homogeneous histopathology according to the 8 histopathological classification subgroups. The sperm retrieval rate was the main outcome.

Testicular spermatozoa were successfully retrieved in 27 men (28%). The sperm retrieval rate in those with heterogeneous histopathology was higher than men with homogeneous histopathology (47% vs 12%; *P < *.001). The sperm retrieval rate of each histopathological subgroup in men who had the heterogeneous histopathology was higher, compared with the homogeneous histopathology (Sertoli cell only [SCO]: 30% vs 6%; maturation arrest [MA]: 38% vs 0%; tubular hyalinization: 42% vs 20%, respectively). Under the optical magnification, the sperm retrieval rate was significantly higher in men with heterogeneous vs homogeneous tubules (65% vs 15%, *P < *.001). Moreover, the sperm retrieval rate of the contralateral testicular was higher in men who had heterogeneous tubules, compared with the homogeneous tubules (25% vs 3%; *P = *.036).

Heterogenicity of histopathology is an effective predictor in men with histopathological information available from a previous diagnostic biopsy or conventional TESE attempt preoperatively for successful sperm retrieval. Homogeneous tubules seem beneficial for some patients to perform a limited (superficial) contralateral micro-TESE after no spermatozoa were identified initially.

## 1 Introduction

Azoospermia affects approximately 1% of males and 10% to 15% of infertile men.^[1]^ Nonobstructive azoospermia (NOA), which is caused by testicular failure, represents 60% of all azoospermia cases.^[2]^ Microdissection testicular sperm extraction (micro-TESE) has become an effective procedure to retrieve spermatozoa in patients with NOA for intracytoplasmic sperm injection, with a high sperm retrieval rate (SRR) and minimal postoperative complications.^[3–5]^

Various studies^[6–8]^ have focused on predicting the presence of spermatozoa in the testis preoperatively. Follicle-stimulating hormone (FSH), luteinizing hormone (LH), and testicular volume have poor predictive value for successful micro-TESE. Paternal age may have an adverse effect on SSR in patients with Klinefelter's syndrome.^[9]^ Histological findings are generally the most useful predictor for successful TESE.^[10,11]^ However, the SRR varies greatly and the possible reason is that most studies had no further identification in histopathological classification.

Only a few studies evaluate the presence of spermatozoa intraoperatively. It is recognized, under optical magnification, those tubules to be identifiable as larger and more opaque or whiter tubules, presumably contains more intratubular germ cells with active spermatogenesis.^[3]^ The intraoperative identification of ≥5 motile and/or nonmotile spermatozoa at the time of unilateral micro-TESE allowed us to correctly limit the surgical procedure to one testicle.^[12]^ Ramasamy et al^[13]^ found only 40 out of the 506 men who underwent bilateral testicular microdissection had sperm found on the contralateral side when no sperm were identified on the initial side. Therefore, it is worth exploring the way to further identify the spermatogenesis focus and reduce the unnecessary hazards.

The aim of the present study is to determine whether the heterogenicity of testicular histopathology and seminiferous tubules can further predict the micro-TESE outcome for NOA patients. Moreover, it further assesses its value of tubules identification at the time of micro-TESE in guiding intraoperative planning.

## 2 Materials and methods

The study protocol was approved by the Ethics Committee of the First Hospital of Jilin University and written informed consent was obtained from all participants.

### 2.1 Study design and patients

The present study is a retrospective analysis of 94 cases with NOA who underwent micro-TESE from 2016 to 2017 in the Reproductive Medicine Center of the First Clinical Hospital of Jilin University. All patients were confirmed to be azoospermia using at least 2 different centrifuged semen analyses according to WHO criteria. All patients performed karyotype and Y chromosomal microdeletion analyses. Around 12 patients had a 47,XXY karyotype and 4 patients had AZFc microdeletions.

Preoperatively identifiable factors, including age, FSH, LH, testosterone, the presence of a varicocele, history of an undescended testis, history of testicular cancer, and history of cryptorchidism. Testis volume was measured at physical examination. The average volume of the both testes was used for analyzed. Patients with proved obstructive azoospermia were excluded. All the procedures were performed by the same surgeon.

### 2.2 Surgical technique

The procedure of micro-TESE has been described previously in detail.^[3]^ Briefly, under general anaesthetic, a midline scrotal incision was made, the tunica vaginalis was opened to expose the tunica albuginea. An equatorial incision was made over the tunica albuginea under an operative microscope (S88, Carl Zeiss Jena, Germany), taking care to avoid vasculature injury. Microdissection was then performed at 15 to 18 magnification under an operative microscope to identify larger and more opaque seminiferous tubules. The specimens were then immediately examined by an embryologist in the operating room. If no spermatozoa were seen intraoperatively, the testicular tissue was thoroughly examined for the presence of spermatozoa by another embryologist to in the embryology laboratory 12 to 24 hour later to avoid missed diagnosis. Tissue specimen was placed in Bouin's solution and sent for histopathological analysis.

### 2.3 Histopathological analysis

**Figure 1:**
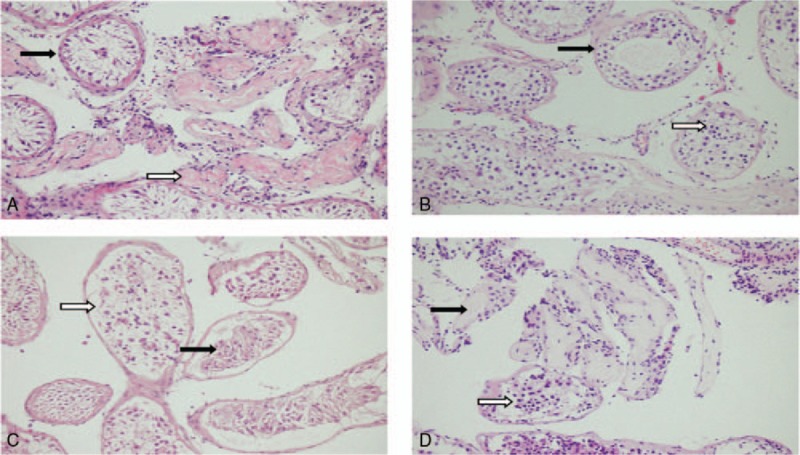
Heterogeneous histopathology consists of 4 patterns: (A) mix or secondary Sertoli cell only (SCO): some seminiferous tubules show Sertoli cell (SC) (black arrow) and some tubules replaced with hyaline substance (white arrow); (B) incomplete maturation arrest (MA): some tubules show arrest of spermatogenesis at the primary spermatocyte stage (black arrow) and some tubules arrest of spermatogenesis at the early spermatid phase (white arrow); (C) partial hypospermatogenesis: tubules show SCO (black arrow) and normal tubules (white arrow); (D) incomplete tubular hyalinization: most tubules hyalinization (black arrow), mixed some germ cells (white arrow). MA = maturation arrest, SCO = Sertoli cell only.

The histopathological information used in this study was from a single random intraoperative biopsy taken at the time of micro-TESE. Base on a previous histopathology pattern,^[14,15]^ we classified testicular histopathology into: complete Sertoli cell only (SCO): The Sertoli cells are of normal number and appearance without hyalinization, incomplete SCO (Fig. 1A): The Sertoli cells are diminished in number and highly altered in shape; in many tubules they are lost and replaced with hyaline substance. Some tubules appear to be completely reabsorbed (ghost tubules).^[16]^ Complete maturation arrest (MA): all the biopsy showed completely MA, either early or late MA, mixed MA (Fig. 1B): a portion of the biopsy revealed an alternate pathology (i.e., Sertoli cell only syndrome) or a biopsy showed both early MA and late MA, complete hypospermatogenesis: all the tubules showed reduction in the number of normal spermatogenetic cells, partial hypospermatogenesis (Fig. 1C): partial tubules showed hypospermatogenesis and others showed an alternate pathology, complete tubular hyalinization: all the tubules were replaced with the hyaline substance, absence of germ cells and Sertoli cells, incomplete tubular hyalinization (Fig. 1D): majority tubules hyalinization, mixed some germ cells/ Sertoli cells. In this study, patients were classified as homogeneous and heterogeneous histopathology: if biopsy with more than one pattern such as incomplete SCO, mixed MA, partial hypospermatogenesis and incomplete tubular hyalinization, we classified that as a heterogeneous pattern; if biopsy contained a single histopathological pattern throughout the sample such as complete SCO, complete MA, complete hypospermatogenesis and complete tubular hyalinization, it is considered a homogeneous pattern.

### 2.4 Diameter of seminiferous tubules

**Figure 2:**
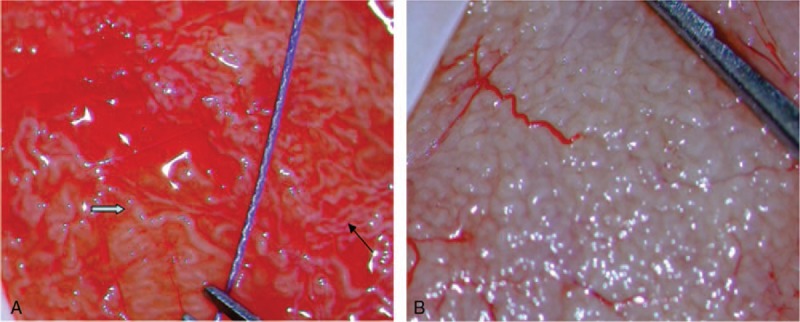
(A) Heterogeneous tubules: the diameter of thick tubule (white arrow) is more than 50 μm larger than that of thin tubule (black arrow); 5/0 surgical suture: diameter of 100 μm. (B) Homogeneous tubules.

Under optical magnification, seminiferous tubules were divided into 2 groups: the tubules were measured using 5/0 surgical suture (Polysorb, Covidien) which had a diameter of 100 μm. If the diameter difference between the most dilated tubules and the finest tubules was less 50 μm (half of 5/0 surgical suture), we classified as homogeneous tubules (Fig. 2B). In contrast, if the diameter difference was 50 μm or greater, we classified as heterogeneous tubules (Fig. 2A). SRR was compared between these 2 groups.

### 2.5 Statistical analysis

All statistical data were analyzed with SPSS, version 17.0 (SPSS Inc.). For quantitative data such as age, testis size, FSH, LH, and testosterone levels, independent-sample t test was used to compare the 2 groups. The qualitative variables such as spermatozoa retrieval rate was evaluated by the chi-square or Fisher's exact test. *P < *.05 was considered statistically significant.

## 3 Result

### 3.1 Sperm retrieval rate

Around 94 patients suffering from NOA with a mean age (range) of 31 (23–46) years were included in the present study. The overall SRR was 29%. The median testicular volume was 6.3 (± 3.3) cc.

### 3.2 Heterogeneous vs homogeneous histopathology

Of the 94 men, 45 patients (48%) were classified as having heterogeneous histopathology, and the rest 49 patients (52%) were classified as having homogeneous histopathology. The characteristic differences between heterogeneous and homogeneous histopathology were showed in Table 1. No significant difference was found in the testicular volume, mean age, FSH and LH. The SRR was higher in patient who had heterogeneous histopathology vs homogeneous histopathology (47% vs 12%; *P < *.001). The SRR of each subgroup in men who had the heterogeneous histopathology was higher, compared with the homogeneous histopathology besides hypospermatogenesis (SCO: 29% vs 6%; MA: 38% vs 0%; tubular hyalinization: 42% vs 20%, hypospermatogenesis: 100% vs 100%, respectively). The testosterone was lower in the heterogeneous group (*P = *.02).

**Table 1:**
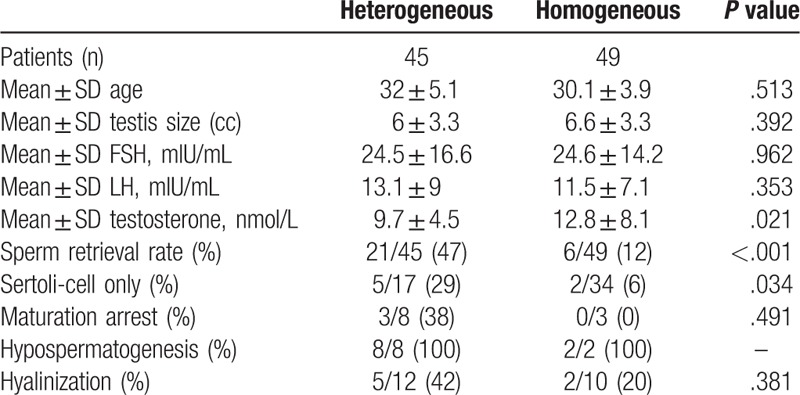
Heterogeneous vs homogeneous histopathology.

### 3.3 Heterogeneous vs homogeneous tubules

Under optical magnification, seminiferous tubules were found to be homogeneous in 68 patients (72%), from which spermatozoa were retrieved in 10 patients (15%). As for the rest 26 patients (28%), the tubules appeared to be heterogeneous, spermatozoa were retrieved in 17 patients (65%). In addition, men with heterogeneous tubules had smaller testicular size (4.9 vs 6.9 cc, *P = *.006), lower testosterone (8.8 vs 12.3 nmol/L, *P = *.006), higher FSH and LH levels (30 vs 22.5 mIU/mL, *P = *.031 and 15.1 vs11.2 mIU/mL, *P = *.034, respectively) (Table 2). Further, of the 73 men who underwent bilateral testicular microdissection sperm were found on the contralateral side in only 5 patients (7%) after no sperm were identified on the initial side. The SRR of the contralateral testicular was higher in men who had heterogeneous, compared with the homogeneous tubules (25% vs 3%; *P = *.036) (Table 2).

**Table 2:**
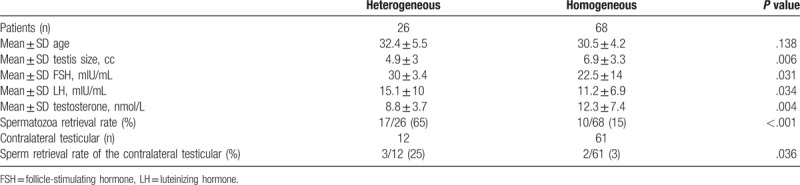
Heterogeneous vs homogeneous tubules under optical magnification.

## 4 Discussion

Microdissection TESE in patients with NOA is an effective technique to reduce the incidence of complications and maximize the spermatozoa retrieval. Amer et al^[5]^ suggested testicular haematoma and permanent echogenic foci were significantly lower in the microdissection side compared with the conventional side one month after TESE. Similar result was found in another study observed a lower rate of haematoma after micro-TESE compared with the conventional TESE one month after surgery.^[17]^ Moreover, Ramasamy et al^[18]^ found a decrease in testosterone levels had also been shown following micro-TESE. Therefore, it is important to explore the way to reduce the hazards and predict the success of micro-TESE.

Previous studies^[10,11]^ had concluded that testicular histology was the best predictor of a successful TESE. Men with hypospermatogenesis pattern were thought have an effective prognosis for successful treatment, with a very high SRR.^[11,19]^ But the SRR in patients with SCO and MA had a wide range from 23% to 43% and from 27% to 75%, respectively.^[11,17,19]^ This possible reason was that most studies had no further identification in histopathological classification. Only a few studies mentioned histopathological heterogenicity,^[5,19]^ but no comprehensive research had been done.

In the present study, we found a significantly higher SRR in men who had incomplete SCO than complete SCO (29% vs 6%, *P = *.034). Anniballo et al. expounded the difference between pure SCO and mixed SCO. No histological alteration can be seen in pure SCO. Men with mixed SCOS the Sertoli cells were altered in number and shape, many tubules were replaced with hyaline substance.^[16]^ Due to the underlying congenital disorder characterized by homogeneous SCO, it was difficult to find spermatozoa. Reduction of both unnecessary TESE and sperm retrieval failure could be achieved by identification in histopathological classification.

In our study, of the 11 men with MA, 3 patients with unsuccessful sperm retrieval were classified as having complete MA. The SRR was 38% for the 8 patients with mixed MA. Similarly, men with incomplete tubular hyalinization had higher SRR than those with complete tubular hyalinization (42% vs 20%). Bernie et al^[20]^ also reported men with late and focal MA have a higher SRR than men with early and diffuse MA (78% vs 40%; 57% vs 35%). This finding may be reasonable, since success of sperm retrieval with NOA depends on finding the better spermatogenesis, which often presents in heterogeneous areas. If all the testicular tissue is homogeneous distribution, spermatozoa are unlikely to be found. Therefore, further identification in histopathological classification is definitely necessary and a good predictor for successful sperm retrieval.

Given that we did not perform conventional TESE on patients with NOA preoperatively, the histopathology used in this study was from intraoperative samples taken during micro-TESE. There is no difference between the samples obtained from micro-TESE and a diagnostic biopsy or conventional TESE. The findings in this study can be used for patients with histopathological information available from a previous diagnostic biopsy or conventional TESE attempt preoperatively, particularly in men with NOA who have normal testis volume and normal FSH.

However, not all the patients performed diagnostic biopsy before micro-TESE, since the invasive procedure may increase the chance of complications. Moreover, we had studied the relationship between the diameter of the tubules and the SRR. Under optical magnification, we found a lower SRR in men who had homogeneous tubules, compared with men who had heterogeneous tubules (15% vs 65%). Although Amer et al reported sperm retrieval rate was significantly higher when seminiferous tubules diameter measured ≥ 300 μm, compared with diameter < 300 μm,^[21]^ not all patients with larger homogeneous tubules mean spermatozoa. Indeed, the purpose of the micro-TESE is to find the heterogeneous area. Intraoperatively, we observed the entire testicular tubules under optical magnification: the more heterogeneous the tubules seen, the more likely there were to have spermatozoa.

In addition, we investigated the SRR of the contralateral testicular after no sperm were identified initially. Ramasamy et al^[13]^ reported up to 8% of those had sperm on the contralateral side. In this study, of the 77 patients who underwent bilateral micro-TESE, 5 (7%) had successful sperm retrieval on the contralateral testicular. The SRR of the contralateral testicular was significantly higher in men who had heterogeneous, compared with the homogeneous tubules (25% vs 3%). Homogeneous tubules can be a good predictor in guiding intraoperative planning. If a homogeneous pattern of tubules is showed under optical magnification, it is feasible to perform a limited (superficial) contralateral micro-TESE to reduce the incidence of complications.

In conclusion, heterogeneous testicular may be more efficient in men with histopathological information available from a previous diagnostic biopsy or conventional TESE attempt preoperatively for predicting successful testicular spermatozoa retrieval. Men with heterogeneous histopathology and tubules had a higher SRR than those who have homogeneous histopathology and tubules. Moreover, intraoperative assessment of homogeneous tubules seems beneficial for patients to perform a limited (superficial) contralateral micro-TESE after no spermatozoa were identified initially, which requires further study to verify.

## Author contributions

Authorship: YY is the first author, collected the sample, and wrote the article; QX was responsible for clinical cases collection and analysis; RW and LL performed the histopathological analysis; HG reviewed genetic analysis; RL performed critical revision of article; YP involved in the critical revision of the article and final approval of article.

**Data curation:** Qi Xi.

**Funding acquisition:** Ruizhi Liu.

**Methodology:** Ruixue Wang, Hongguo Zhang, Leilei Li.

**Writing – original draft:** Yang Yu.

**Writing – review & editing:** Yuan Pan.
